# Development and reliability of the histological THROMBEX-classification rule for thrombotic emboli of acute ischemic stroke patients

**DOI:** 10.1186/s42466-021-00149-6

**Published:** 2021-09-20

**Authors:** Julika Ribbat-Idel, Florian Stellmacher, Florian Jann, Nicolas Kalms, Inke R. König, Marcus Ohlrich, Georg Royl, Stefan Klotz, Thomas Kurz, Andrè Kemmling, Florian C. Roessler

**Affiliations:** 1 Institute of Pathology, University of Lübeck, Ratzeburger Allee 160, 23538 Lübeck, Germany; 2 Institute of Pathology, Research Center Borstel - Leibniz Lung Center, 23845 Borstel, Germany; 3 Department of Neurology, Justus-Liebig-University Gießen, Klinikstraße 33, 35385 Gießen, Germany; 4 Institute of Medical Biometry and Statistics, University of Lübeck, Ratzeburger Allee 160 (House 24), 23562 Lübeck, Germany; 5 Department of Neurology, Sana Kliniken Lübeck GmbH, Kronsforder Allee 71-73, 23560 Lübeck, Germany; 6 Department of Neurology and Center of Brain, Behaviour and Metabolism, University of Lübeck, Ratzeburger Allee 160, 23538 Lübeck, Germany; 7 Department of Cardiovascular and Thoracic Surgery, Segeberger Kliniken, Am Kurpark 1, 23795 Bad Segeberg, Germany; 8 Department of Internal Medicine II/Cardiology, Angiology, and Intensive Care Medicine, University of Lübeck, Ratzeburger Allee 160, 23538 Lübeck, Germany; 9 Department of Neuroradiology, Westpfalz-Klinikum, Hellmut-Hartert-Straße 1, 67655 Kaiserslautern, Germany

**Keywords:** Ischemic stroke, Histological classification, Thrombotic emboli, Etiology

## Abstract

**Background:**

Thrombus histology has become a potential diagnostic tool for the etiology assessment of patients with ischemic stroke caused by embolic proximal vessel occlusion. We validated a classification rule that differentiates between cardiac and arteriosclerotic emboli in individual stroke patients. We aim to describe in detail the development of this classification rule and disclose its reliability.

**Methods:**

The classification rule is based on the hypothesis that cardiac emboli arise out of separation thrombi and arteriosclerotic emboli result from agglutinative thrombi. 125 emboli recovered by thrombectomy from stroke patients and 11 thrombi serving as references for cardiac (n = 5) and arteriosclerotic emboli (n = 6) were Hematoxylin and eosin, Elastica-van Gieson and CD61 stained and rated independently by two histopathologists blinded to the presumed etiology by several pre-defined criteria. Intra- and interobserver reliabilities of all criteria were determined. Out of the different criteria, three criteria with the most satisfactory reliability values were selected to compose the classification rule that was finally adjusted to the reference thrombi. Reliabilities of the classification rule were calculated by using the emboli of stroke patients.

**Results:**

The classification rule reached intraobserver reliabilities for the two raters of 92.9% and 68.2%, respectively. Interobserver reliability was 69.9%.

**Conclusions:**

A new classification rule for emboli obtained from thrombectomy was established. Within the limitations of histological investigations, it is reliable and able to distinguish between cardioembolic and arteriosclerotic emboli.

**Supplementary Information:**

The online version contains supplementary material available at 10.1186/s42466-021-00149-6.

## Background

Cardiac and arteriosclerotic emboli are the most frequent causes of proximal vessel occlusion of acute ischemic stroke patients [1]. However, in many cases, these pathomechanisms remain undetectable or there are indications for both cardiac and arteriosclerotic emboli. This leads to uncertainty concerning secondary prevention and increasing risks for further strokes [2].

In thromboembolic ischemic strokes, pathophysiological considerations, as well as results of recent clinical trials, suggest that the histology of emboli indicates the underlying thrombogenesis [3–7]. Thus, determining the histological characteristics of emboli may reveal their formation process to further specify secondary prevention after strokes with uncertain genesis.

The prospective, blinded THROMBEX trial addresses this clinically relevant issue. Histological features of emboli extracted from acute ischemic stroke patients were examined and correlated with the medical history and diagnostic workup to develop the first histological classification rule that can distinguish between cardiac and arteriosclerotic emboli in individual stroke patients. Its prediction accuracy is in the range of 70–78% [8].

The classification rule is based on the following pathophysiological considerations:

The rigidity and strength of a clot increase with more extensive and denser distribution of platelets [9] and fibrin net [4, 10–12]. It is expected that older clots show a denser and more compact structure with increased amounts of dissolved neutrophil granulocytes and dissolved red cells. Within minutes after clot formation, neutrophils penetrate the thrombus reaching their highest concentration after a few hours before they start to disintegrate. Further penetration into the more compact and fibrin-rich clot becomes increasingly difficult. Therefore, older clots contain smaller proportions of intact neutrophils and red cells [13, 14].

It is commonly assumed that agglutinative thrombi result primarily from hemodynamic impairment around reduced blood flow velocities [15, 16]. Accordingly, the histological composition of the resulting thrombus is smooth and loose with only a little organized structure. In consequence, “red clots” with a fine fibrin net, few and locally restricted platelets, and many intact neutrophil granulocytes and red cells would arise in the venous vessels. However, agglutinative thrombi also emerge in areas of high blood flow velocities as the tail part of a mixed-thrombus. Usually, the head section of the mixed-thrombus is a separation thrombus firmly attached to a damaged arterial vessel wall [6, 14, 17, 18].

Separation thrombi generally originate in the area of endothelial damage [18–23]. These clots (described as “white clots” due to their low concentration of hemoglobin) contain dense fibrin nets, many and widely distributed platelets, as well as many shattered neutrophils and red cells [18, 19, 21, 23, 24]. By histological quality, they are dense, compact, rigid, stable, and appear older like already organized clots. In the course of many cardiac diseases, the endocardium is frequently damaged. Presumably, those endothelial lesions are mostly the reason for clot formation within heart cavities. There is evidence that endothelial dysfunction is the primary underlying pathology that directly triggers clot formation with the side effect of atrial fibrillation and hemodynamic changes in the auricles [25, 26]. Activation of coagulation factors and platelets, inflammatory processes as well as structural and functional myocardial remodeling are additional factors in the cascade of clot formation [27–30].

**Fig. 1:**
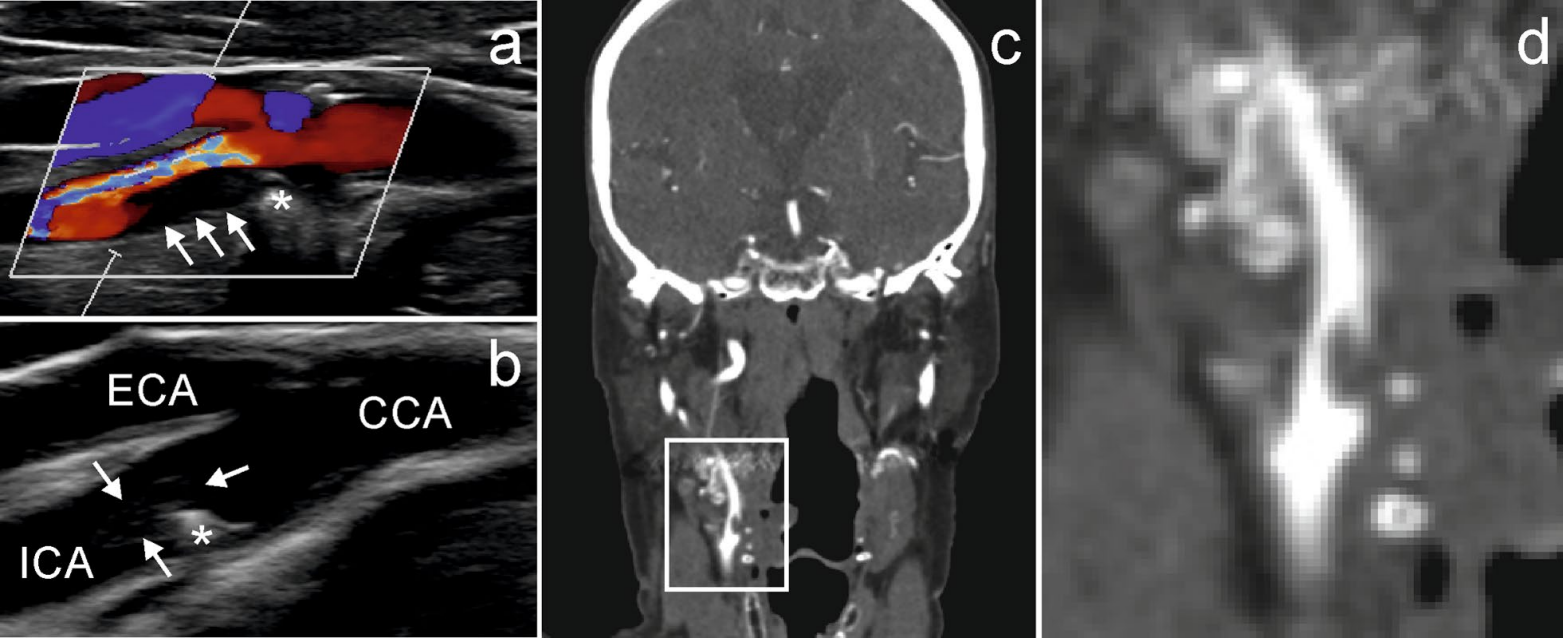
Example of an agglutinative thrombus **a**: Duplex sonography outlined stenosis (*) in the right ICA of a 90-year-old woman suffering from multiple embolic strokes located in the ipsilateral cerebral hemisphere. A hypoechogenic structure was attached to the stenosis (arrows). **b**: In the course of several control examinations, the structure shifted into the lumen of the ICA. **c**: This finding was confirmed by CT-angiography. **d**: Detail enlargement of picture **c**. The patient underwent a thromboendarterectomy and an agglutinative thrombus was extracted. CCA: common carotid artery; ECA: external carotid artery; ICA: internal carotid artery

Due to these reasonable and profound results of prior investigations, we hypothesize that:

1. Cardiac emboli usually arise out of separation thrombi caused by an endothelial dysfunction of the heart. Longer dwell times in the heart cavity caused by associated atrial fibrillation and reduced blood flow velocity enable increased clot organization with progressive cell death, further local thrombin activation, accumulation of fibrin, and increased cross-linking. The resulting (“white”) clots contain dense fibrin nets, many platelets, and many shattered neutrophil granulocytes and red cells. They become harder and more resistant before they are carried away by the blood flow causing a stroke.
2. Arteriosclerotic emboli usually result from agglutinative tail parts of mixed-thrombi or clotting processes in the slipstream of stenosis. Those (“red”) clots contain only fine fibrin nets, few and locally restricted platelets, and many intact neutrophils and red cells. They have torn away soon after formation by fast streaming blood and flow turbulences. Therefore, they are smooth and loose and their structure is poorly organized. Figure 1 shows such a typical agglutinative thrombus that caused several embolic strokes.

s

The defined histological classification rule refers to these hypotheses and is suitable to differentiate between cardiac and arteriosclerotic emboli [8], which are the most frequent causes of proximal vessel occlusion of acute ischemic stroke patients [1].

We already published the validation of this classification rule [8]. However, sufficient reproducibility is also indispensable for its future application by different scientists or for repeated investigations performed by the same histopathologist. Therefore, we aim to reveal the intra- and interobserver reliabilities of all histological criteria that were available for our classification rule and describe the development steps of the classification rule that are based on these reliability values. Finally, we present the intraobserver and interobserver reliabilities of the final classification rule.

The THROMBEX-classification rule might contribute to specifying secondary prevention in cases of strokes with large vessel occlusions of uncertain genesis but indications of cardiac or arteriosclerotic emboli.

## Methods

For the development of the classification rule, both hypotheses had to be transferred into reliable histological criteria. For this, we calculated the intraobserver and interobserver reliabilities of all available histological criteria. Only the most reliable criteria were chosen to formulate the dichotomous classification rule. Then, its cut-off values were defined by an adjustment to reference thrombi. Finally, the reproducibility of the final classification rule was determined by emboli obtained from stroke patients.

### Acquisition of reference thrombi and thrombotic emboli

Eleven clots of certain origin served as reference thrombi. Five clots were withdrawn out of left-sided heart cavities during cardiosurgical interventions. They were considered to be cardioembolic clots. Six clots represented arterioembolic clots as they were extracted from coronary arteries during cardiac catheter examinations. Relevant patient data are listed in Table 1.

**Table 1:** Baseline characteristics of patients from which the reference thrombi were obtained

Baseline characteristics	post stenotic thrombi of coronary arteries (N = 6)	thrombi from left-sided heart cavities (N = 5)
Age [y] mean, range	60.5, 38—77	65.8, 56–76
Female sex, n (%)	0 (0)	2 (40)
Vascular risk factors		
Arterial hypertension, n (%)	3 (50)	4 (80)
Diabetes mellitus, n (%)	1 (16.7)	2 (40)
Hyperlipoproteinemia, n (%)	1 (16.7)	1 (20)
History of smoking, n (%)	2 (33.3)	0 (0)
Atrial fibrillation, n (%)	2 (33.3)	1 (20)
Premedication		
ASS, n (%)	6 (100)	4 (80)
ASS + ticagrelor or clopidogrel, n (%)	0 (0)	2 (40)
Heparin, n (%)	6 (100)	0 (0)
Anticoagulation, n (%)	0 (0)	2 (40)

Three of the six post-stenotic thrombi originate from the right coronary artery. The remaining three thrombi were found in the anterior interventricular artery. Concerning the thrombi from left-sided heart cavities, one was extracted from the left atrial appendage. All the others were located in the left ventricle

To determine the reliability of the final classification rule, 125 thrombotic emboli were collected from acute ischemic stroke patients who underwent thrombectomy. In all cases, mechanical thrombectomy was performed by a standardized coaxial procedure with stent retriever systems (Trevo XP, 4 × 20 mm or 6 × 25 mm, Stryker, Fremont, USA).

### Histological examination

Samples were formalin-fixed (4% buffered formalin, BÜFA, Hude, Germany) and paraffin-embedded (FFPE, Paraffin, Leica Microsystems, Wetzlar, Germany).

2 µm thick slices were cut from the paraffin blocks by microtome and placed on Super Frost glass slides (Menzel, Braunschweig, Germany). Hematoxylin and eosin (HE) stains (Merck, Darmstadt, Germany) and Elastica-van Gieson (EvG) stains (Merck, Darmstadt and Waldeck/Chroma, Münster, both Germany) were carried out. Immunohistochemistry was performed using the PostBlock HRP-Polymer and ZytoChem Plus Kit (both Zytomed, Berlin, Germany). The CD61 (Zytomed, Berlin, Germany) antibody was diluted at 1:250 and pretreated by steamer. Serial sections were stained and arranged on a single microscope slide. Thus, the clot was screened over a wide range before a representative section was selected for further evaluation.

**Fig. 2:**
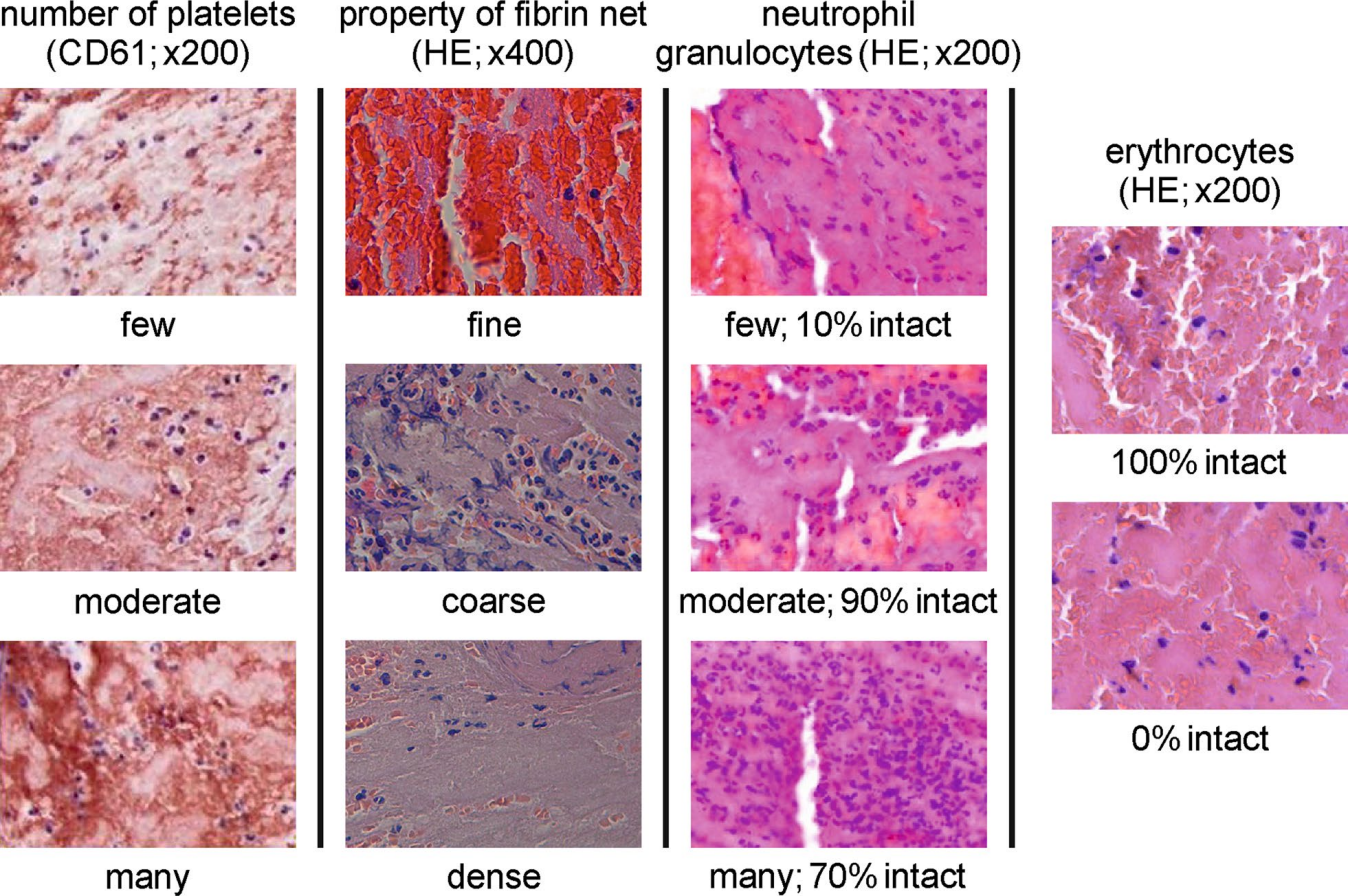
Synopsis of all histological criteria and their characteristic features The number of platelets is determined by immunohistochemical staining (CD61). The property of the fibrin net is determined by hematoxylin–eosin (HE) and Elastica-van Gieson staining (EvG). The number of neutrophil granulocytes, as well as the proportion of intact neutrophils and red cells to the total number of the respective blood cells, is classified by HE. The number of platelets and neutrophils is described by categorical scale using the characteristic features “few “, “moderate “, and “many “. Continuous percentage scales are used to specify the property of fibrin net subdivided in the characteristic features “fine “, “coarse “, and “dense “ and to define the proportions of intact neutrophils and red cells to their total numbers. Following the terms of cloud formations, the distribution of platelets is described as “stratus “, “cirrus “, or “cumulus “. Vivid pictures are published in [8]. The histological cross-sections were captured with 200-fold original magnification. Only the fibrin net was pictured with 400-fold original magnification

During a training period, both histopathologists randomly selected 15 cases to find a consensus and to agree on standards. Secondly, a synopsis of all histological criteria and their characteristic features was created (Fig. 2). Then, all HE, EvG, and CD61 stains were examined by the two raters (rater I and II) independently using Olympus BX50 microscope or Carl Zeiss Axioskop 40 microscope, both with fluorite objectives with plano-correction. Both raters were blinded concerning the total number and the etiology of the thrombi, as well as to the clinical diagnosis of the stroke patients and their demographics and treatment.

The CD61 stains were used to assess platelets. On the one hand, their amount was categorically measured ranging “few”, “moderate” and “many”. On the other hand, their distribution was described using an analogy to basic meteorological cloud shapes of the World Meteorological Organization published in the International Cloud Atlas [31].

Thus, platelet aggregation was subdivided into “stratus”, “cirrus”, and “cumulus” [8]. Following the wording of the International Cloud Atlas, in a stratus distribution platelets occur as a merged layer with a fairly uniform appearance or in the form of ragged patches. A cirrus distribution is characterized by detached clusters in the form of delicate filaments or narrow bands, having a hair-like appearance. Cumulus-like arranged platelets appear as detached and dense clusters with sharp outlines. They may be in the form of domes or towers. Sometimes they are ragged. Upon microscopic evaluation, the platelet distribution was interpreted and continuously assigned to the three patterns according to their quantitative occurrence in percentage.

Reading the HE and EvG stains, histopathologists reviewed the fibrin net regarding its quality and assigned percentage values to each of the three features”fine”, “coarse”, and “dense”. Furthermore, based on the HE stains the number of neutrophil granulocytes was measured using a categorical scale ranging “few”, “moderate”, and “many”. Finally, neutrophils and red cells were examined assessing their integrity, dividing them up into wholesome or shattered. Continuous percentage scales were used to specify the proportions of intact neutrophils and red cells to their total numbers.

### Reliability of individual histological criteria

The quality of a criterion is reflected by its reproducibility. Only criteria with a sufficiently high interobserver and intraobserver reliability were considered for the histological classification rule. Therefore, intraobserver and interobserver reliabilities of all criteria and their characteristic features were determined using all available clots, the reference thrombi (N = 11) as well as the emboli extracted from stroke patients (N = 125). During transportation, the microscope slides of three reference thrombi were damaged so badly that they were lost for further evaluation. Therefore, rater II had only access to eight of the eleven reference thrombi (three from left-sided heart cavities and five from coronary arteries).

To determine intraobserver reliability, both raters were requested to repeat their assessment of clot composition after six months in 23 cases. Hindsight bias was prevented by blinding both raters to this procedure. Therefore, they were not aware that they had already evaluated these clots previously. Caused by faulty long-term archiving repeat evaluation was performed by rater I for only 14 clots. Caused by preterm fading of tissue stainings rater II could only determine platelet distribution in 22 cases and the number of platelets in 21 cases.

To determine interobserver reliability, eight reference thrombi and 125 emboli extracted from acute stroke patients were available. Staining errors caused preterm fading of tissue stainings in different HE, EvG, and CD61 stains for nine of these 133 clots. Therefore, the number of cases varies between 127 and 132 for the different histological assessments.

### Specification of the cut-off values

Cut-off values were adjusted until the maximum number of reference thrombi were correctly assigned by the classification rule to their pathophysiological formation process.

### Reliability of the final classification rule

The reliabilities of the final classification rule were determined by using all available emboli of the 125 stroke patients. Reference thrombi were not suitable because they were already used for result-oriented adjustment of the classification cut-off values.

To determine intraobserver reliability, repeated histological analyses were carried out unknowingly at an interval of six months by rater I and rater II. Both raters should re-examine 23 clots. Caused by the above-mentioned faulty long-term archiving and staining errors, rater I classified only 14 and rater II only 22 emboli.

Due to staining errors and consequent incomplete data sets, assessments of only 123 emboli from stroke patients were available to determine the interobserver reliability.

A coherent description of the caseload and the reasons for data loss can be found in the Additional file 1.

### Calculations and statistics

To assess interobserver and intraobserver reliability of categorical variables, Cohen’s kappa coefficients were estimated with 95% confidence intervals. For continuous variables, we estimated Cronbach’s alpha. The agreement between and within raters is shown using Bland–Altman plots estimating the mean difference with 95% confidence intervals. Analyses were performed using R version 3.5.0 with the packages psych (version 1.7.2) and BlandAltmanLeh (version 0.3.1) [32].

## Results

### Practicability of the classification method

The classification is based on default staining and common examination material and is therefore not expensive. Complete histological evaluation of one clot required only a few minutes for an incorporated histopathologist.

### Reliability of the single histological criteria

Table 2 contains results for the intraobserver and interobserver reliabilities. All characteristic features with satisfactory reliability values are highlighted in italics.

**Table 2:** Statistical data of the intra- and interobserver reliabilities of the different histological criteria

Intra- and interobserver reliability			
Rater (N)	Few	Moderate	Many
Number of platelets: κ [95% CI]			
R1 (14)	*−* 0.12 [*−* 0.31 to 0.07]	0.39 [*−* 0.08 to 0.87]	*0.58 [0.05*–*1.00]*
R2 (21)	*0.7 [0.31*–*1.00]*	*0.52 [0.16*–*0.87]*	*0.7 [0.4*–*1.00]*
R1–R2 (130)	*0.51 [0.32*–*0.7]*	0.23 [0.10–0.36]	0.19 [0.04–0.34]

Rater (N)	Stratus	Cirrus	Cumulus
Distribution of platelets: α [mean diff.; limit: lower–upper]			
R1 (14)	*0.89 [0.19; − 0.18 to 0.55]*	0.56 [*−* 0.19; *−* 0.75 to 0.36]	*0.79 [0.01; − 0.44 to 0.46]*
R2 (22)	*0.87 [− 0.02; − 0.46 to 0.42]*	*0.81 [0.06; − 0.41 to 0.53]*	*0.84 [− 0.04; − 0.47 to 0.39]*
R1–R2 (127)	*0.62 [− 0.07; − 0.69 to 0.55]*	0.56 [*−* 0.01; *−* 0.59 to 0.56]	*0.62 [0.08; − 0.52 to 0.68]*

Rater (N)	Fine	Coarse	Dense
Property of fibrin net: α [mean diff.; limit: lower–upper]			
R1 (14)	*0.91 [0.01; − 0.25 to 0.27]*	*0.80 [− 0.01; − 0.33 to 0.30]*	*0.92 [0.01; − 0.28 to 0.30]*
R2 (23)	*0.66 [− 0.14; − 0.59 to 0.31]*	0.57 [0.15; *−* 0.35–0.65]	*0.73 [− 0.01; − 0.50 to 0.47]*
R1–R2 (131)	*0.73 [0.07; − 0.36 to 0.51]*	0.45 [*−* 0.01; *−* 0.49 to 0.47]	*0.8 [− 0.07; − 0.46 to 0.33]*

Rater (N)	Few	Moderate	Many
Number of neutrophil granulocytes: κ [95% CI]			
R1 (14)	*0.66 [0.25*–*1.00]*	0.29 [*−* 0.21–0.78]	0.43 [*−* 0.10–0.96]
R2 (23)	0.38 [0.03–0.72]	0.47 [0.13–0.81]	*0.64 [0.27*–*1.00]*
R1–R2 (132)	0.38 [0.20–0.56]	0.22 [0.06–0.39]	0.35 [0.18–0.52]
Number of intact neutrophil granulocytes: α [mean diff.; limit: lower–upper]			
R1 (14)	*0.71 [0.03; − 0.28 to 0.34]*		
R2 (23)	0.43 [0.02; − 0.65 to 0.68]		
R1–R2 (132)	*0.65 [− 0.03; − 0.47 to 0.41]*		
Number of intact red cells: α [mean diff.; limit: lower–upper]			
R1 (14)	*0.94 [0; − 0.23 to 0.22]*		
R2 (23)	*1.00 [0; − 0.04 to 0.04]*		
R1–R2 (132)	*0.64 [0.06; − 0.48 to 0.60]*		

R1, intraobserver reliability for rater I; R2, intraobserver reliability for rater II; R1-R2, interobserver reliability. The related number of cases is indicated in parentheses. Weighted Cohen’s kappa (κ) was calculated for categorically scaled criteria. CI = confidence interval. For the continuously scaled criteria, Cronbach’s alpha is used. The related Bland–Altman plots can be seen in the Additional file 1: Figs. S1, S2, S3. Numerical values indicating sufficient reliability are written in italics. Chosen tolerance levels: κ = 0.5; α = 0.6.

κ < 0 = poor, 0 ≤ κ ≤ 0.2 = slight, 0.2 < κ ≤ 0.4 = fair, 0.4 < κ ≤ 0.6 = moderate, 0.6 < κ ≤ 0.8 = substantial, and 0.8 < κ ≤ 1 = (almost) perfect agreement.

α < 0.5 = unacceptable, 0.5 ≤ α < 0.6 = poor, 0.6 ≤ α < 0.7 = questionable, 0.7 ≤ α < 0.8 = acceptable, 0.8 ≤ α < 0.9 = good, and 0.9 ≤ α = excellent.

We decided to choose tolerance levels for reproducibility following published values, although the reproducibility of different studies cannot be compared directly. Most kappa values concerning histological classifications are in the range of 0.41–0.6 (moderate agreement). The defined tolerance levels (κ: 0.5; Cronbach’s alpha: 0.6) were often exceeded by the calculated values of the present histological criteria.

Additional file 1: Figs. S1, S2, S3 depict Bland–Altman graphs for all characteristic features of continuously scaled criteria. In all cases, the mean of the difference was a horizontal line close to the zero lines. Therefore, the systematic bias of the measurements was small. Furthermore, the plot suggests that differences between the two raters were independent of the amount of the determined values. The systematic bias of the measurement was constant over the entire range of values without any drift. Due to the small number of cases, standard deviations were high. Only criteria with the most satisfactory reliability values were chosen for the classification rule. They included:

1. Platelet distribution (CD61)
2. Property of fibrin net (HE and EvG)
3. Proportions of intact neutrophils and red cells to their total number (HE)

### Development of the classification rule

As discussed in the introduction section we set up two hypotheses based on pathophysiological considerations and results gained by experimental and clinical studies of other research groups:

1. Cardiac emboli comply with separation thrombi with dense fibrin nets, many platelets, and small proportions of intact neutrophils and red cells. These clots are dense, compact, rigid, stable, and appear older.
2. Arteriosclerotic emboli are equivalent to agglutinative thrombi with fine fibrin nets, few platelets, masses of red cells, and high proportions of intact neutrophils and red cells. These clots are smooth, unstable, and appear younger.

Considering the chosen criteria for reliable investigations, we formulated the classification rule as follows:

The histological structure of an embolus points to an arterioembolic formation process if two of the following three criteria are met:

1. Proportion of dense fibrin ≤ 30%.
2. Platelet distribution meets cumulus criterion ≥ 90%.
3. Proportion of intact neutrophils and red cells ≥ 80%.

**Fig. 3:**
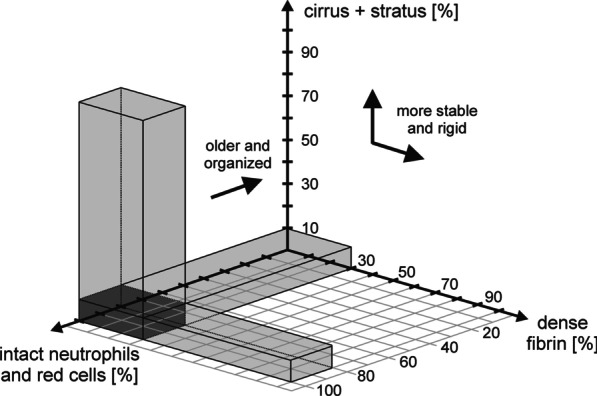
Graphical illustration of the classification rule within the three-dimensional parameter space A clot will be classified as “ARTERIO” (arterioembolic formation process) if its characteristic features are located within the grey shaded coordinate space. Otherwise, the clot will be evaluated as “CARDIO” (cardioembolic formation process)

Such smooth, unstable, and recent emboli are defined as “ARTERIO” (Fig. 3).

Otherwise, histological classification points to a cardioembolic formation process (“CARDIO”) leading to dense, compact, and old-looking emboli.

The cut-off values were determined by an optimization process. They were adjusted until the maximum number of reference thrombi were correctly assigned by the classification rule to their pathophysiological formation process.

### Reliability of the final classification rule

All values concerning the reliability of the final classification rule determined by the evaluation of emboli from acute stroke patients are listed in Table 3. Intraobserver reliabilities for the two raters reached values of 92.9% and 68.2%, respectively. Interobserver reliability was 69.9%. Due to small sample numbers, the confidence intervals for Cohen’s kappa are large.

**Table 3:** Statistical results concerning the histological evaluation of the reference thrombi and the emboli of stroke patients

Reference thrombi R1 (N = 11)/R2 (N = 8)	Histological evaluation	
	CARDIO	ARTERIO
Certain genesis of thrombi		
Cardiac (C)	4/3	1/0
Arteriosclerotic (A)	2/1	4/4
Intraobserver reliability [%, (95% CI)]		
C: R1 (N = 5)/R2 (N = 3)	80 (28.4–99.5)/100 (29.2–95.0)	
A: R1 (N = 6)/R2 (N = 5)	66.7 (22.3–95.7)/80 (28.4–99.5)	
C + A: R1 (N = 11)/R2 (N = 8)	72.7/87.5	
Interobserver reliability (N = 8)		
Agreement [%]	87.5	
κ (95% CI)	0.75 (0.31–1)	
Emboli of stroke patients		
Intraobserver reliability [%/κ (95% CI)]		
R1 (N = 14)	92.9/0.81 (0.46–1)	
R2 (N = 22)	68.2/0.36 (− 0.02 to 0.75)	
Interobserver reliability (N = 123)		
Agreement [%]	69.9	
κ (95% CI)	0.39 (0.23–0.55)	

R1, rater I; R2, rater II; κ, Cohen’s kappa; CI, confidence interval

## Discussion

The developed classification rule is based on two hypotheses. As described in the introduction section, these hypotheses are supported by many other investigations.

The successful adjustment to reference thrombi and its successful validation [8] are further arguments for our presented classification rule.

Our results contradict the traditional concept that recent red thrombi mainly originate from low flow regions, as has been postulated for cardiac emboli [33], while white thrombi, existing mostly out of platelets and a high amount of fibrin, usually arise in regions of fast-moving blood, as in arteriosclerotic emboli from large arteries [15, 16]. However, concerning the composition of cardiac emboli, our hypothesis is supported by Wysokinski et al. who concluded that all cardiac emboli consist mainly out of fibrin, platelets, and debris [23]. Furthermore, they found that embolized thrombi show twice as many platelet-rich domains compared to non-embolized atrial thrombi.

The developed classification rule is reliable. However, the reliabilities of histological evaluations are lower than the corresponding values of other diagnostic methods.

Allison et al. defined three overall root causes of diagnostic variability of histopathological investigations: pathologist-related, study methodology-related, and specimen-related [34].

### Pathologist-related causes of diagnostic variability

Emboli retrieved by thrombectomy consist of complex, heterogeneous, and anisotropic materials. To avoid considerable errors, in our investigations serial sections were stained and arranged on a single microscope slide. Thus, the clot was screened over a wide range before a representative section was selected for further evaluation. However, a complete evaluation of an embolus is unfeasible and different raters might choose different sections for their final evaluation. Therefore, much of the interobserver variation stems from an interpretation based on operator experience and opinion. Already at the beginning of our study rater I had outstanding professional experience whereas rater II received training at that time. Accordingly, compared with rater I the intraobserver reliability is lower for rater II undergoing a learning process. On completion of the learning process agreement between both raters was nearly 70% in using the final classification rule for emboli of stroke patients (Table 3).

### Study methodology-related causes of diagnostic variability

Sufficient reliability can only be achieved by precise definitions, unambiguous terminology, and simple classification rules. The Bland–Altman plots (Additional file 1: Figs. S1, S2, S3) point out that for all characteristic features of continuously scaled criteria rating in the medium range is most difficult. This means that professional opinion can become critical in ambiguous cases. Both, slight excess and undercutting the cut-off values can lead to completely different results of our dichotomous classification. Therefore, it might be appropriate in the future to categorize a case as borderline and to provide a differential diagnosis. An alternative approach would be the optimization of the chosen histological criteria.

For the intraobserver reliability, the length of the interval between the repeated examinations is crucial. In our study, the interval accounts for an adequate length of six months.

### Specimen-related causes of diagnostic variability

Poor stain quality, limited tissue quantity, or artifacts account for the majority of specimen-related diagnostic variability. As already mentioned in the methods section, in a few cases the quality of sample material was too low for an assessment of all histological criteria by both raters. However, the vast majority of specimens were suitable even for repeated analysis in the course of six months indicating the feasibility of the developed classification.

According to the literature known to us, reproducibility values are rarely determined for histological classifications and there are no generally accepted thresholds. Even common and useful histological classifications and grading systems are limited concerning their reliability reaching kappa values in the range of 0.41–0.6 (signifying moderate agreement) [35–41]. Considering this, the results obtained by our classification rule are very acceptable.

### Further specific limitations

Each classification has its certain drawbacks. In our case these are:

1. The low number of reference thrombi. No significant correlation between histological evaluation and etiology of reference thrombi was achieved (Table 3). Higher sample numbers could probably improve the adjustment of the cut-off values.
2. The used reference thrombi are not cerebral emboli. Only the clots that were extracted from left-side heart cavities might embolize into the brain. The clots that were extracted from coronary arteries do not need to be equal to arteriosclerotic emboli that usually originate from the internal carotid artery (ICA). However, Silvain et al. described the layered aspect of coronary thrombi according to a mixed-thrombus consisting of parts with high fibrin and platelet proportions and parts with entrapped red cells and inflammatory cells [14]. The presumption that clots obtained from arteriosclerotic altered coronary arteries are similar to arteriosclerotic emboli of the ICA is further supported by the theory that transition from a stable to an unstable lesion of coronary arteries results from intraplaque hemorrhage causing instability with rupture and re-hemorrhage in the ulcerated atherosclerotic plaques [18]. This essential role of red cells in the pathogenesis of coronary artery disease was confirmed in carotid artery disease by a large pathological study, which showed that recent plaque hemorrhage had occurred in all patients who suffered a recent cerebral stroke [42].
3. The histological composition of the reference thrombi might be affected by multiple parameters. The main ones are listed in Table 1. In particular, medical treatment before symptom onset may play a decisive role. The adjustment of the classification rule might be influenced by this but not the values of reproducibility.
4. How the classification rule was formed from the single histological criteria might have been different. For example, a weighting of the histological criteria could have been applied. Currently, classification limits are perpendicular to each other (Fig. 3). This is certainly not physiological.
5. Only two different pathophysiological types of clots are considered by the classification rule. Obviously, the reality is much more complex. However, in the majority of cases, acute embolic strokes with proximal vessel occlusion are caused by cardiac or arteriosclerotic emboli [1]. Furthermore, the both largest studies in this field performed by Boeckh-Behrens et al. [43] and Sporns et al. [7] showed, on the one hand, a strong histological overlap of cryptogenic and cardiac emboli and on the other hand, a strong overlap of arteriosclerotic emboli and emboli of other determined causes. Therefore, from the histological and the etiological point of view this simplification seems to be acceptable.

However, it has been proven that the classification rule is reliable and can sufficiently differentiate the underlying etiology of the 11 reference thrombi even if this was in the course of a result-oriented adjustment. The validity of the classification rule concerning the evaluation of stroke etiology of individual patients is presented in another publication [8]. In this publication, there is also a comparison with other studies on histological classifications of thrombotic emboli. In summary, most of the studies, in particular the both largest studies [7, 43], correspond with our developed classification rule [8]. However, all previous studies are based on group comparison. In contrast, our classification rule was developed to enable inferences about stroke causes in individual patients, which is vitally important to improve secondary prevention of patients with embolic strokes of unknown source. In a recent study, Kim et al. developed the BOCS_2_ scale based on histopathological and angiographic criteria to distinguish between cerebral emboli caused by large artery atherosclerosis or cardioembolism [44]. The findings of Kim et al. support our hypothesis that arteriosclerotic emboli result from agglutinative tail parts of mixed-thrombi. Although the BOCS_2_ scale is based on a descriptive comparison and has not yet been validated, we believe that this combination of different investigation methods is a very promising approach. Presumably, it is time to reach a consensus with other working groups regarding the most promising study for a multicenter trial.

## Conclusions

To our knowledge, this is the first attempt to develop a histological classification rule to differentiate between cardiac and arteriosclerotic cerebral emboli through well-justified pathophysiological considerations, results of prior investigations, and adjustment to reference thrombi before specimens are examined.

For further improvement, several limitations need to be overcome. However, the first results concerning the reproducibility and validity of the final classification rule are promising.

In the future, this classification rule might help to improve secondary prevention of patients with embolic strokes of an unknown source.

## Supplementary Information

**Additional file 1**. Caseload and the reasons for data loss; Bland-Altman plots of the continuously scaled criteria.

## Data Availability

The datasets used and/or analyzed during the current study are available from the corresponding author on reasonable request.

## Abbreviations

CI: Confidence interval
EvG: Elastica-van Gieson
HE: Hematoxylin and eosin
ICA: Internal carotid artery
κ: Cohen’s kappa
