# Supplementary material for: Development and reliability of the histological THROMBEX-classification rule for thrombotic emboli of acute ischemic stroke patients
Source: Neurol Res Pract. 2021 Sep 20;3:50. doi: 10.1186/s42466-021-00149-6 (PMC8451083; doi:10.1186/s42466-021-00149-6)
Supplement: Supplementary file 1 — Additional file 1. Caseload and the reasons for data loss; Bland-Altman plots of the continuously scaled criteria. [file 42466_2021_149_MOESM1_ESM.pdf]

SUPPLEMENTAL MATERIAL

Development and reliability of the histological THROMBEX-classification rule for thrombotic emboli of acute ischemic stroke patients

Julika Ribbat-Idel, MD[1] and Florian Stellmacher, MD[2]; Florian Jann[3]; Nicolas Kalms[3]; Inke R. König, PhD[4]; Marcus Ohlrich[5]; Georg Royl, MD[6]; Stefan Klotz, MD[7]; Thomas Kurz, MD[8]; Andrè Kemmling, MD[9]; Florian C. Roessler, MD, PhD[3]

[1] Institute of Pathology, †

[2] Institute of Pathology, Research Center Borstel - Leibniz Lung Center, 23845 Borstel, Germany.

[3] Department of Neurology, Justus-Liebig-University Gießen, Klinikstraße 33, 35385 Gießen, Germany.

[4] Institute of Medical Biometry and Statistics, University of Lübeck, Ratzeburger Allee 160 (House 24), 23562 Lübeck, Germany.

[5] Department of Neurology, Sana Kliniken Lübeck GmbH, Kronsforder Allee 71-73, 23560 Lübeck, Germany.

[6] Department of Neurology and Center of brain, behaviour and metabolism †

[7] Department of Cardiovascular and Thoracic Surgery, Segeberger Kliniken, Am Kurpark 1, 23795 Bad Segeberg, Germany

[8] Department of Internal Medicine II / Cardiology, Angiology, and Intensive Care Medicine, †

[9] Department of Neuroradiology, Westpfalz-Klinikum, Hellmut-Hartert-Straße 1, 67655 Kaiserslautern, Germany.

† University of Lübeck, Ratzeburger Allee 160, 23538 Lübeck, Germany.

Address for correspondence:

Dr. med. Dr. rer.-medic. Dipl.-Phys. Florian C. Roessler

Klinik und Poliklinik für Neurologie, Universitätsklinikum Standort Gießen, Klinikstraße 33, 35385 Gießen, Germany.

Telephone: 0049-(0)641-985-56814.

E-mail: florian.roessler@neuro.med.uni-giessen.de

Description of the caseload and the reasons for data loss

In our study, we used in total 136 clots consisting of 11 reference thrombi and 125 thrombotic emboli from acute ischemic stroke patients. The two independent histological raters worked at different locations. Therefore, the samples were sent several times back and forth. During transportation, the microscope slides of three reference thrombi were damaged so badly that they were lost for further evaluation. Therefore, rater II only evaluated eight of eleven reference thrombi. Accordingly, only 133 clots remained for the determination of the interobserver reliability.

Staining errors caused preterm fading of tissue stainings in different HE, EvG, and CD61 stains for nine of these 133 clots. Therefore, the number of cases varies between 127 and 132 for the different histological assessments. For the same reason, only 123 of 125 clots were available to determine the interobserver reliability concerning the final classification of the emboli of stroke patients.

For the determination of the intraobserver reliability, each rater should re-examine 23 clots after six months. Due to an archiving error, rater I could only access 14 of 23 clots. Caused by preterm fading of tissue stainings rater II could only determine the number of platelets in 21 cases and platelet distribution in 22 cases, leading to a final classification of only 22 clots.

Summarized, we had problems regarding transport (3/136), staining errors (9/133), and archiving for a long period (9/23).

Transport and long-term archiving are specific problems of our reliability study and should not play a role when clots will be investigated immediately in the future clinical routine. Staining errors remain a source of error for the application of the classification rule, which can only be avoided by working very carefully.

Supplemental Figures

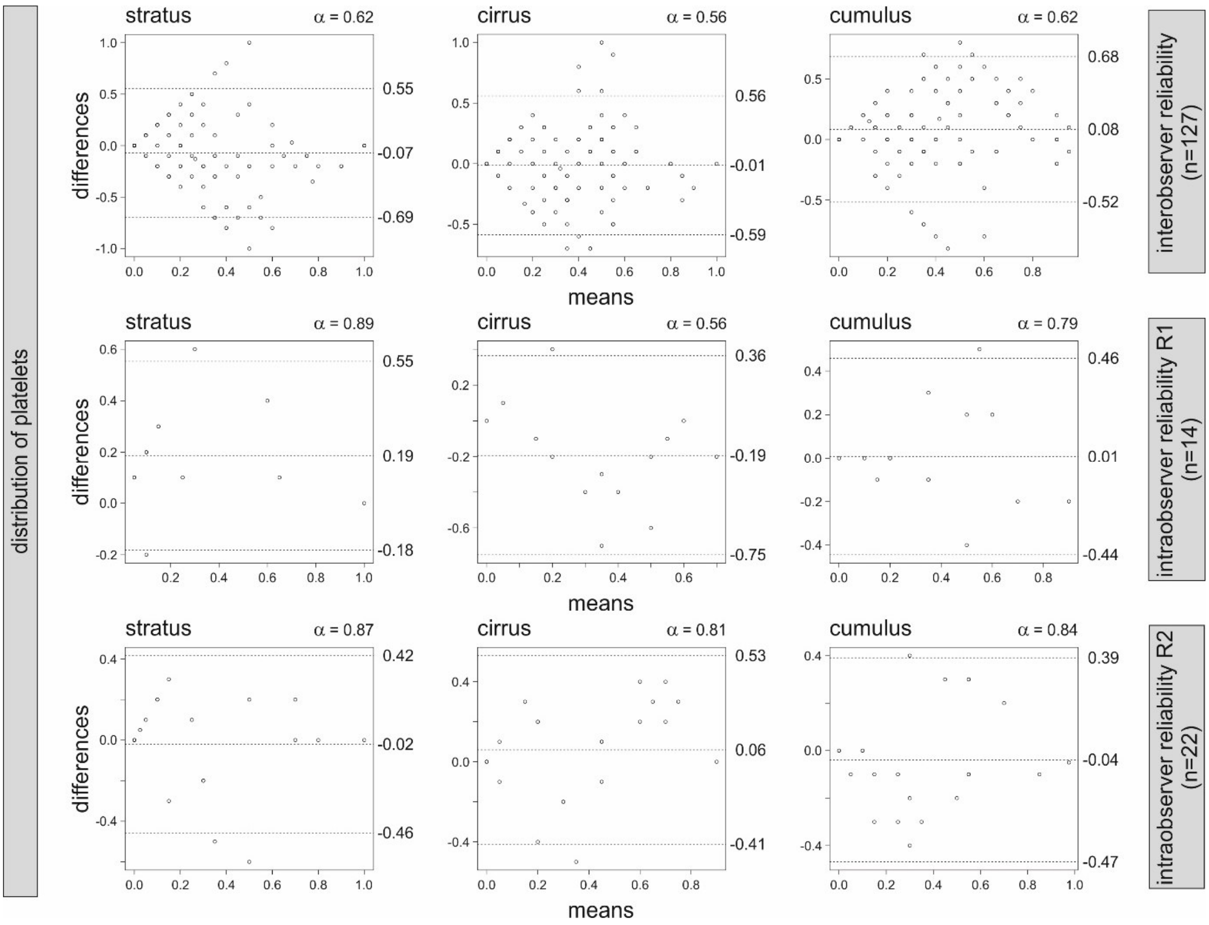

Fig. 1 Bland-Altman-plot for the intra- and interobserver reliability concerning the distribution of platelets For every characteristic feature (“stratus”, “cirrus”, and “cumulus”) of the platelet distribution the intra- and interobserver reliability of the two histopathologists (R1 = rater I; R2 = rater II) are illustrated by depicting the difference of each of two related measurements A and B as a function of its mean-value (1/2(A+B)). In the case of intraobserver reliabilities, A and B are results of two independent measurements performed by the same histopathologist. In the case of interobserver reliabilities, A and B are results of two independent measurements performed by each of the two histopathologists. The middle horizontal line displays the mean of the difference (A-B). It is a measure for the systematic bias of the measurement. The other two parallel lines correspond to the mean ± 1.96 standard deviations. With a probability of 95%, the variance of two histological assessments lies in between these lower and upper limits. α = Cronbach’s alpha.

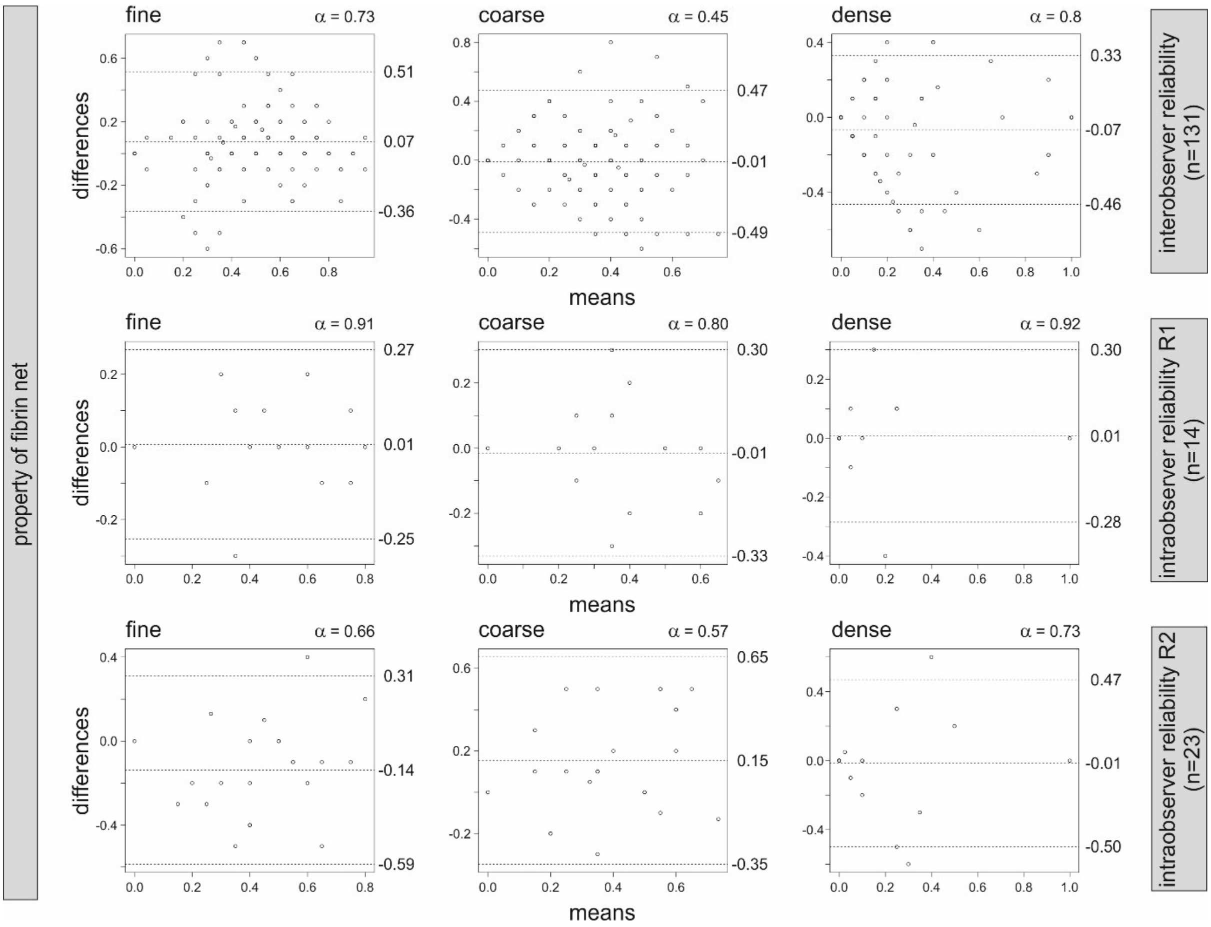

Fig. 2 Bland-Altman-plot for the intra- and interobserver reliability concerning the property of fibrin net All graphs are presented in the same way as described in Figure I.

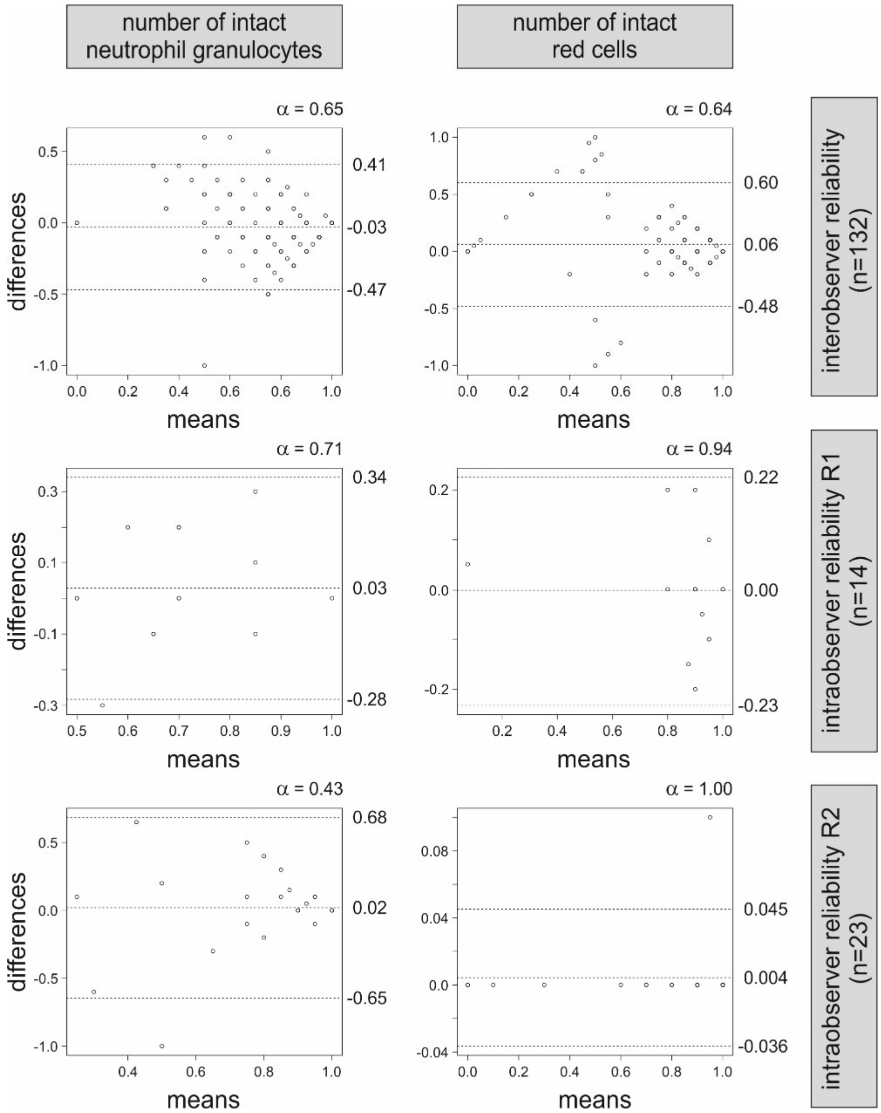

Fig. 3 Bland-Altman plot for the intra- and interobserver reliability concerning the number of intact neutrophil granulocytes and of intact red cells All graphs are presented in the same way as described in Figure I.
